# The Effect of Antibiotics on the Eradication of Multidrug-Resistant Organisms in Intestinal Carriers—A Systematic Review with Meta-Analysis

**DOI:** 10.3390/antibiotics13080747

**Published:** 2024-08-09

**Authors:** Maja Johanne Søndergaard Knudsen, Ingrid Maria Cecilia Rubin, Andreas Munk Petersen

**Affiliations:** 1 Department of Clinical Microbiology, Copenhagen University Hospital–Amager and Hvidovre, 2650 Hvidovre, Denmark; maja.johanne.soendergaard.knudsen@regionh.dk (M.J.S.K.);; 2 Gastrounit, Medical Section, Copenhagen University Hospital-Amager and Hvidovre, 2650 Hvidovre, Denmark; 3 Department of Clinical Medicine, University of Copenhagen, 2200 Copenhagen, Denmark

**Keywords:** VREfm, CPE, ESBL, eradication, decolonisation, antibiotics

## Abstract

Objectives: The aim of this systematic review was to investigate the effect of antibiotics on the eradication of multidrug-resistant organisms (MRO) in intestinal carriers. We defined multidrug-resistant organisms as vancomycin-resistant *Enterococcus faecium* (VREfm), and multidrug-resistant Gram-negative *Enterobacterales.* Methods: We searched the EMBASE, Cochrane Central, and PubMed databases from inception to medio November 2023. We included randomised and controlled clinical trials (RCTs), that investigated the effect of antibiotics on the eradication of multidrug-resistant organisms in intestinal carriers. Finally, we performed a meta-analysis. Results: We included five RTCs in the systematic review. In four studies an effect of antibiotics on the eradication of MRO was shown at the end of intervention, but it was not sustained at follow-up. In the fifth study, the effect at the end of intervention was not reported, and there was no observed effect of the intervention at follow-up. We included four studies in the meta-analysis, and it suggests an effect of antibiotics on the eradication of MRO in intestinal carriers at the end of follow-up with a *p*-value of 0.04 (95% confidence interval 1.02–1.95). None of the studies reported a significant increase in resistance to the study drug. Gastrointestinal disorders were the most frequent non-severe adverse event. Conclusions: The effect of antibiotics on the eradication of multidrug-resistant organisms in intestinal carriers was not statistically significant in any of the five included studies; however, we found a significant effect in the pooled meta-analysis. As the confidence interval is large, we cannot determine the clinical importance of this finding, and it should be further investigated.

## 1. Introduction

In this systematic review we aimed to determine the effect of antibiotics on the eradication of multidrug-resistant organisms (MRO), defined as vancomycin-resistant *Enterococcus faecium* (VREfm), multidrug-resistant Gram-negative *Enterobacterales*, Extended-Spectrum Beta-Lactamase producing *Enterobacterales* (ESBL-E), and Carbapenemase-Producing *Enterobacterales* (CPE), in intestinal carriers. All the MRO can cause both intestinal colonisation and invasive infections. A previous study focusing on VREfm found an association between the VREfm clones that the patients were colonised with and the VREfm clones they subsequently developed infection with [1]. In a large prospective observational study of patients from the haematology/oncology departments, the authors showed that previous colonisation with ESBL-E was identified as the most important risk factor for a subsequent ESBL-E bloodstream infection [2]. Considering these results along with the challenges of treating invasive infections with MRO, we assume it would be beneficial to eradicate MRO from the intestine.

Antimicrobial resistance is an increasingly severe health threat worldwide. According to the surveillance report, Antimicrobial Resistance Surveillance in Europe, published in 2023 (2021 data), 15 European countries reported at least 25% of their invasive *Klebsiella pneumoniae* isolates to be carbapenem-resistant [3]. Furthermore, it has been estimated that in 2019, 1.27 million deaths were directly associated with antimicrobial resistance, and the Review on Antimicrobial Resistance predicts that this number will increase to 10 million by 2050 [4,5]. In addition to the rise in antimicrobial resistance, the use of antibiotics worldwide has increased by 65% from 2000 to 2015 [6]. Invasive infections with MRO often require treatment with last-resort antibiotics that are critically important.

Antibiotic stewardship is important to avoid inappropriate and unnecessary use of antibiotics. The keys of antibiotics stewardship are to use the right antibiotic treatment for the specific infection in the correct dosage and duration [7]. In 2017, the Danish Ministry of Health published the National Action Plan on Antibiotics in Human Healthcare. The action plan contained three goals to reduce the antibiotic consumption, which were as follows: (i) reduction in the number of antibiotic prescriptions; (ii) reduction in the use of critically important antibiotics; and (iii) to use narrow-spectrum antibiotics instead of broad-spectrum antibiotics when possible [8]. Therefore, if antibiotics are used to eradicate MRO in intestinal carriers, the advantage, e.g., reduction in the number of invasive infections with MRO, should outweigh the disadvantages. Antibiotic stewardship poses a challenge in the treatment of infections caused by MRO, e.g., ESBL-E. Traditionally, carbapenems have been a frequently used treatment for infections with ESBL-E, and this practice is thought to be one of the causes of the emergence of CPE [9].

Another contributor to the increasing prevalence of MRO is spread in health care facilities. Among MRO, VREfm and CPE in particular are pathogens that are well known to cause outbreaks in hospital wards [10,11,12,13,14]. To prevent these outbreaks, hygiene measures and detection of carriers are important. In the Capital Region of Denmark, we use contact isolation precautions for all admitted patients with VREfm or CPE invasive infections and/or intestinal carriage. Furthermore, we have an active surveillance programme, and in case of outbreaks in hospital wards, we perform weekly screening of all patients in the ward [13].

We hypothesised that eradication of MRO from the intestine would prevent invasive infections with MRO over time. We aimed to identify placebo-controlled Randomised Clinical Trials (RCTs) that have included intestinal carriers of MRO and investigated the effect of an antibiotic intervention on eradication of MRO intestinal carriage. As secondary outcomes, we investigated whether the eradication intervention contributed to further development of resistance, and we determined the number of invasive infections with MRO in both arms of antibiotic intervention and control group. Finally, we looked at serious and non-serious adverse events.

## 2. Results

### 2.1. Article Selection

**Figure 1:**
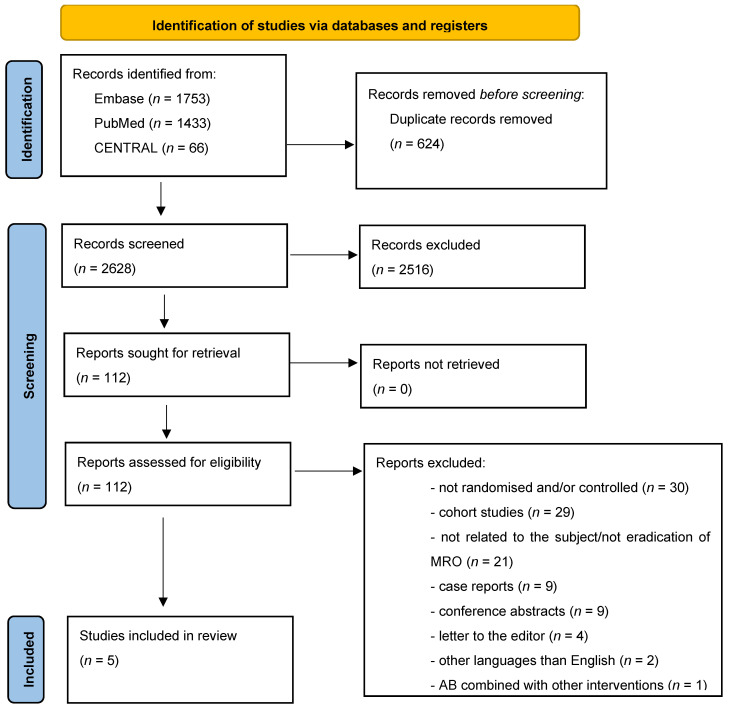
An overview of the selected articles. The figure is adapted from the PRISMA statement. *From*: Page MJ, McKenzie JE, Bossuyt PM, Boutron I, Hoffmann TC, Mulrow CD, et al. The PRISMA 2020 statement: an updated guideline for reporting systematic reviews. BMJ 2021;372:n71. doi: 10.1136/bmj.n71.

We identified 3252 articles in PubMed, EMBASE, and Cochrane Central databases. Of these articles, 624 articles were duplicates and removed automatically by the Covidence computer program. In the initial screening process, we went through 2628 titles and abstracts of articles, and we selected 111 articles for the thorough full-text evaluation. During the full-text evaluation we found five articles eligible for inclusion in the systematic review [15,16,17,18,19]. The remaining articles were excluded due to lack of randomisation, cohort study design, articles not related to the study subject or articles not reporting eradication of MRO, case reports, conference abstracts, letters to the editor, combination of multiple interventions, articles in languages other than English, review articles, and clinical trial protocols. An overview of the selection of articles can be found in Figure 1, adapted from the PRISMA statement [20].

### 2.2. Description of Included Studies

The five included articles were RCTs, three of which were double-blinded and placebo-controlled [15,16,17]. Two were non-blinded, and did not provide a placebo intervention to their control group [18,19]. The RCTs were conducted from 2012 to 2021 in Israel [15], Switzerland [16], Belarus [18], Germany [17], and Spain [19]. In total, 290 patients were included in the 5 studies. The participants had a mean age from 49 years to 71.6 years. In two studies, the included patients were considered immunocompromised [17,18], and in one study the included patients were undergoing solid organ transplantation [19]. All studies included participants that were carriers of Gram-negative bacteria with different mechanisms, e.g., ESBL-E and CPE. We were not able to include any studies that enrolled carriers of VREfm. One study was underpowered and failed to include the anticipated number of participants [17]. In the study by Saidel-Odes and colleagues, the authors included hospitalised intestinal carriers of CPE, and they do not report a power size calculation [15].

The duration of the antibiotic intervention varied from 7 to 14 days. All studies used oral administration of antibiotics. One study used an oropharyngeal gel consisting of colistin and gentamicin, along with the oral solution of gentamicin and polymyxin E [15]. Another study used colistin in monotherapy [18]. Two studies used colistin in combination with neomycin [16,19], and one study used colistin in combination with gentamicin and fosfomycin [17]. One study administered oral nitrofurantoin for five days to their subgroup of participants with ESBL-E bacteriuria [16]. The follow-up period varied from 21 to 42 days. All studies used culture-based methods to detect MRO. In addition to culture, one study performed PCR to detect extended-spectrum beta-lactamases, carbapenemases, and plasmid-mediated AmpC [19]. Please refer to Table 1 for the description of all included articles.

### 2.3. Effect of Antibiotics on Eradication of Multidrug-Resistant Organisms

Four studies investigated the number of participants with eradication of MRO at the end of intervention. In all of those studies, a significant effect of antibiotics on the eradication of MRO was shown at the end of intervention, but was not sustained at follow-up [15,16,17,18]. In the fifth study, the authors did not report the eradication of MRO at the end of intervention. At the time of follow-up, they did not find a significant effect of antibiotics on the eradication of MRO [19]. Throughout all five included studies, the intervention with antibiotics did not show a significant effect on the eradication of MRO in intestinal carriers.

### 2.4. Meta-Analysis

We included four studies in the meta-analysis [16,17,18,19,22]. The fifth study was excluded, as the authors reported the results in percentages, and we could not extract absolute numbers for participants with and without eradication of MRO at follow-up [15].

**Figure 2:**
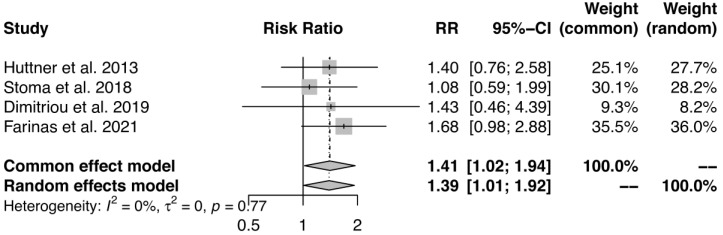
The forest plot from the meta-analysis [16,17,18,19].

The pooled meta-analysis of the four studies suggests an effect of antibiotics on eradication of MRO in intestinal carriers with a *p*-value of 0.04 (95% confidence interval 1.02–1.95). The forest plot is shown in Figure 2, and the funnel plot is shown in Supplementary Figure S1.

We included the same four studies in the NNT analysis, and the result was an NNT of 7.86.

### 2.5. Secondary Outcomes

#### 2.5.1. Development of Resistance

All the included studies performed surveillance of the susceptibility patterns of their chosen study drug. In one study, 3/49 participants in the intervention group, and 1/50 participants in the control group developed resistance to colistin (*p* = 0.618) [19]. Four studies did not observe an increase in resistance to the study drug during the study period [15,16,17,18]. In one of those studies, carbapenem-resistant *Klebsiella pneumoniae* was discovered in one participant in the intervention group, and two participants in the control group [17].

#### 2.5.2. Invasive Infections with MRO

In the study by Dimitriou and colleagues, where they included severely immunocompromised participants from the haematological and oncological departments, 17/18 participants in the intervention group and 10/11 participants in the control group received antibiotic treatment during the study period. Most of these participants received antibiotic treatment due to febrile neutropenia [17]. In the study by Huttner and colleagues, five participants in the control group and one participant in the intervention group received treatment with antibiotics that were efficient against ESBL-E during the study [16]. In two studies, the authors reported no significant change in the risk of invasive infection by the end of their follow-up period [18,19]. In the study by Stoma and colleagues the incidence of bloodstream infections had decreased in the intervention group compared with the control group (3.2% and 12.9% respectively) at 30 days’ follow-up, but it evened out at 90 days’ follow-up [18]. In one study, the authors did not compare the incidence of invasive infection between the two groups [15].

#### 2.5.3. Adverse Events

Gastrointestinal disorders were the most frequent non-severe adverse events. Three studies observed liquid stools in their study population, both in the intervention and in the control groups, with most events in the intervention groups [16,18,19]. In the study by Dimitriou and colleagues, the authors observed non-severe adverse events in eight participants in the intervention group, and in six participants in the control group. Among these adverse events, gastrointestinal disorders were the most frequent. In this study, one participant in the intervention group died due to pneumonia and sepsis [17]. Liquid stools are a well-known adverse event in patients treated with antibiotics due to disturbance to the intestinal microbiota [23]. In the study by Saidel-Odes and colleagues, they observed no non-severe adverse events; however, in this study, five participants in the intervention died, as well as four participants in the control group. Of these, four suffered from pneumonia, three from sepsis, one from cryoglobulinemia, and one died due to cardiac reasons. None of the participants with a fatal outcome had bacteraemia due to carbapenem-resistant *Klebsiella pneumoniae* [15].

### 2.6. Risk of Bias

In general, the included studies had a low risk of bias. Naturally, the two unblinded studies that did not administer a placebo intervention to their control group had a high risk of bias concerning “Concealment of treatment allocation”, “blinding of participants and providers”, and “blinding of people assessing outcome” [18,19]. Three studies had high or unclear risk of bias regarding “drop out of 20% or less”, and “differential drop-out rate” [15,16,17]. Four studies had high or unclear risk of bias in the criterion of “adherence and compliance by treatment group” [15,17,18,19]. One study had unclear risk of bias, and one study had high risk of bias regarding the criterion of “power and sample size” [15,17]. An overview of the results from the risk of bias analysis can be found in Supplementary Figure S2.

## 3. Discussion

In this systematic review and meta-analysis, we have determined the effect of antibiotics on the eradication of MRO in intestinal carriers. We performed a search of studies in PubMed, EMBASE, and Cochrane central databases, and we included five RCTs in the systematic review. Four studies were included in the meta-analysis. Regarding the effect of antibiotics on eradication of MRO, none of the five included studies proved a statistically significant effect at the time of follow-up. The four studies that investigated the effect of antibiotics on the eradication of MRO at the end of intervention all showed a significant effect at this time point. However, this effect was not sustained at follow-up. The results of our meta-analysis showed a significant effect of antibiotics on the eradication of MRO. Nevertheless, with a rather broad 95% confidence interval of 1.02–1.95, we cannot fully determine the clinical relevance of this finding. In addition, found a NNT of 7.86, which we find too high to implement an intervention with antibiotics to routinely eradicate carriers of MRO. Concerning the development of resistance to the administered type of antibiotic, the eradication regimes with antibiotics did not seem to significantly increase resistance in the study populations. In four studies, the authors did not note an increase in resistance, and in the fifth study three participants in the intervention group as opposed to one participant in the control group, developed resistance to colistin [19]. Concerning adverse events, gastrointestinal disorders were the most frequent. Gastrointestinal disorders are well-known side effects of treatment with antibiotics.

Concomitant treatment with antibiotics due to invasive infections is an important limitation and a possible confounder in studies investigating the effect of antibiotics on eradication of MRO. Four of the included studies reported concomitant treatment with antibiotics [16,17,18,19]. In the study by Dimitriou and colleagues, the majority of participants received antibiotics due to febrile neutropenia. In two studies a subgroup of participants received antibiotic treatment for infections with their MRO [16,19]. In the study by Stoma and colleagues, they administered empiric antibiotics in cases of blood stream infections. Additionally, participants with neutropenia received prophylaxis with trimethoprim-sulfamethoxazole against *Pneumocystis jirovecii* [18]. More studies would be required to accurately assess the effect of concomitant treatment with antibiotics during the study period.

Our systematic review with meta-analysis is also limited by the small number of included clinical trials. Furthermore, one of the trials is underpowered, and another trial report no defined power calculations [15,17]. The strength of our study is the clearly defined in- and exclusion criteria. We made the decision to only include studies with a control group to avoid natural decolonisation as a confounder.

In all the included studies, the authors use one rectal swab or one faecal sample from each participant to determine MRO colonisation at the follow-up visits. This is a common method in clinical trials; however, multiple samples are often required in cases of de-isolation of former MRO carriers, who are admitted to hospital. Concerning the effect of antibiotics on the eradication of MRO that was shown in four of the included studies by the end of intervention, the diagnostical method constitutes a limitation. In these studies, culture methods were used to detect MRO, and the antibiotic intervention has undoubtedly entailed a suppression of *Enterobacterales* in the intestine [15,16,17,18]. In the study where the authors use PCR to detect genotypic resistance, no MRO assessment was performed by the end of intervention [19].

In recent years, there has been an increased focus on MRO and the intestinal microbiome. In a very interesting study by Kang and colleagues, they investigated the faecal microbiome in carriers of CPE. The authors examined the microbiome over time in the CPE carriers and compared this to the microbiome from non-CPE carriers in the same households. The found the lowest diversity of the microbiome in the CPE carriers compared to the non-CPE carriers. The diversity of the microbiome in the CPE carriers increased towards the time of decolonisation and reached the same level of diversity as the non-CPE carriers two months after decolonisation [24]. These findings suggest manipulation of the microbiome as possible eradication regimens in carriers of MRO. Successful eradication of MRO after faecal microbiota transplantation (FMT) was first reported in single-patient case reports [25,26]. Following these case reports, the effect of FMT on eradication of MRO in intestinal carriers has been investigated in smaller studies with no control group, and the effect remains to be further determined in larger randomised and controlled clinical trials [27,28,29].

An alternative to active eradication attempts in carriers of multidrug-resistant organisms is studying the rate of natural decolonisation. The time to decolonisation varies in the literature. Concerning VREfm, the natural decolonisation after six months of carriage is reported to be 50–90% [30,31]. In a Parisian cohort from two hospitals with carriers of VREfm and CPE, the authors found that natural decolonisation had happened in 48.2% of patients after 90 days of carriage [32]. Bar-Yoseph and colleagues performed a systematic review with meta-analysis, where they investigated the natural decolonisation of CPE and ESBL-E in carriers, in residents from health care facilities. They found the decolonisation rate to be 23.3% after 1 month, and 64.8% after 12 months. They found no difference between carriers of ESBL-E and carriers of CPE. In the subgroup of ESBL-E carriers in the community, the rates were higher; 52.3% after 1 month, and 74.6% after 12 months [33].

The development of antibiotic resistance to the study drugs was one of our concerns regarding antibiotics for the eradication of MRO, but it did not seem to pose a major challenge in the studies included in this systematic review and meta-analysis. However, we would not recommend the use of antibiotics for the eradication of MRO in intestinal carriers based on the results of this systematic review and meta-analysis. The significant effect of antibiotics on the eradication of MRO found in the meta-analysis has a large 95% confidence interval, as well as a NNT of 7.86, which bring uncertainty to clinical applications. Our perception aligns with the “ESCMID-EUCIC clinical guidelines on decolonization of multidrug-resistant Gram-negative bacteria carriers” in which the authors do not recommend systematic decolonisation of carriers [34]. This is supported by a more recent scoping review by Mascolo and colleagues [35], and a retrospective study by Rieg and colleagues [36].

## 4. Methods

### 4.1. Protocol

We registered the systematic review protocol on PROSPERO “https://www.crd.york.ac.uk/prospero/ (accessed on 4 December 2023)”, and the protocol can be found with the identifier CRD42023489575.

### 4.2. Search Strategy

We performed the search for articles in EMBASE, Cochrane Central, and PubMed databases from inception to medio November 2023. We defined antibiotics as antibiotic, antibacterial, anti-bacterial, neomycin, colistin, polymyxin, gentamicin, paromomycin, ceftazidime, meropenem, imipenem, ertapenem, linezolid, daptomycin, tobramycin, ceftazidime-avibactam, and tigecycline. We defined multidrug resistance as vancomycin resistance, antibiotic resistance, extended spectrum beta lactamase, carbapenemase producing, carbapenemase resistant, multidrug resistance, ESBL-E, CPO, CPE, CRE, CRKP, KPC, carbapenem resistance, carbapenem resistant, VRE, VREfm, and carbapenemase producing. We defined the organisms as Enterococcus faecium, E faecium, Enterococci, Enterobacteriaceae, Enterobacterales, Escherichia coli, E coli, Klebsiella, Klebsiella pneumoniae, Klebsiella oxytoca, K oxytoca, K pneumoniae, Gram negative bacteria, Enterobacteria, and Enterobacteriae. Finally, we defined eradication as Eradication, decolonisation, decolonization, decontamination, and selective digestive decontamination. The full search strategies for the three databases are shown in Supplementary Document S1.

### 4.3. Data Collection

IMCR and MJSK independently performed the initial screening of titles and abstracts. We used the computer program Covidence “https://www.covidence.org (accessed on 13 November 2023)” to manage all extracted articles from the search. The initial screening was succeeded by a full-text evaluation and analysis by IMCR and MJSK independently. Any disagreements between IMCR and MJSK in the two-step screening process were settled by the third author, AMP.

From the included articles, we collected data on author, year of publication, country, study design, intervention (type of antibiotic, dosage, and duration), as well as MRO eradication by the end of the intervention and at follow-up. For the secondary outcomes we extracted data on the development of further resistance during the trial, number of invasive infections with MRO, time of follow-up, and adverse events.

### 4.4. In- and Exclusion of Studies

In this systematic review, we only included studies investigating the effect of antibiotics on the eradication of MRO intestinal carriers. The MRO that we included in the study were VREfm, and multidrug-resistant *Enterobacterales,* e.g., ESBL-E and CPE. We included randomised controlled trials that reported eradication of MRO intestinal carriers as an outcome. We included studies with or without a placebo intervention for their control group.

We excluded animal studies, laboratory studies, articles not related to the subject, articles in other languages than English, review articles, retrospective or prospective cohort studies, case reports, conference abstracts, letters to editors, and guidance documents. We made the decision to exclude studies that combined antibiotics with other interventions, e.g., faecal microbiota transplantation (FMT), if they had no intervention arm with antibiotics alone, as this makes the effect of antibiotics itself unclear.

### 4.5. Meta-Analysis

We conducted a meta-analysis to determine our primary outcome—the effect of antibiotics on the eradication of multidrug-resistant organisms—at the time of follow-up. We performed the statistical analysis in R version 4.2.2 [37]. We used the Mantel–Haenszel method to estimate the risk ratio (RR), and applied both fixed- and random-effects models. The analysis of heterogenicity was insignificant (*p* = 0.77), therefore we used the common effects model in the further analysis. To verify the model, we made a funnel plot.

Finally, we calculated the Number Needed to Treat (NNT) based on the incidence of decolonisation in the intervention and control groups, respectively. We divided one by the difference between the incidence of decolonisation in the intervention and control groups.

### 4.6. Risk of Bias Assessment

To assess the risk of bias in the included RCTs, we used a modified version of the Quality Assessment Tool from the National Institutes of Health “https://www.nhlbi.nih.gov/health-topics/study-quality-assessment-tools, (accessed on 13 March 2024, Supplementary Document S2)”. We modified criteria 9 and 13 to incorporate compliance with the protocol, and no subgroup analysis, respectively. Furthermore, we added the new criteria 15 and 16 to assess the specification of duration of treatment, and the specification of the type and dosage of antibiotic used in the intervention. IMCR and MJSK independently assessed the risk of bias for each included study and reported low risk, high risk, or unclear risk of bias. The third author, AMP, resolved any disagreements between IMCR and MJSK.

## 5. Conclusions

None of the five studies included in our systematic review found a significant effect of antibiotics on the eradication of MRO by the time of follow-up. None of the studies reported a significant increase in resistance to the study drug. Gastrointestinal disorders were the most frequent non-severe adverse event. Our meta-analysis of four of the included studies did show a significant effect, but with a large 95% confidence interval, which brings uncertainty to the clinical application of this intervention. Furthermore, we calculated an NNT of 7.86. Based on this systematic review and meta-analysis, we would not recommend antibiotics for the eradication of MRO in intestinal carriers.

## Figures and Tables

**Table 1:** Describes all included articles.

Paper	Study Design	Intervention (AB and Dose) and Duration	Number of Participants (AB:Placebo)	Mean Age in Years (Range)	Follow-Up	Number of Cleared Individuals at the End of Intervention	Number of Cleared Individuals at the End of Follow-Up
Saidel-Odes et al. [15] 2012 Israel	Randomised (1:1), double-blinded and placebo-controlled study.	Oropharyngeal gel (colistin and gentamicin, 0.5 g 4× daily), oral gentamicin (80 mg 4× daily) and polymyxin E (1 × 106 IU 4× daily) for 7 days.	20:20 Carriers of Carbapenem- Resistant *Klebsiella* *pneumoniae.*	71.6 in the intervention group, 66.5 in the placebo group.	6 weeks	61.1% of patients in the intervention group, and 16.1% of patients in the placebo group.	58.5% of patients in the intervention group, and 33.3% in the placebo group.
Huttner et al. [16] 2013 Switzer-land	Randomised (1:1), double-blinded placebo-controlled parallel-group study.	Oral colistin (1.26 × 106 IU 4× daily) and neomycin (178 mg 4× daily) for 10 days. In case of ESBL-E bacteriuria oral nitrofurantoin was added (100 mg 3× daily) for 5 days.	27:27 Carriers of ESBL-E.	51 (38–67) in intervention group, 61 (48–69) in the placebo group.	28 days	17/25 in the intervention group, 6/26 in the placebo group.	14/27 in the intervention group, 10/27 in the placebo group.
Stoma et al. [18] 2018 Belarus	Randomised (1:1), non-blinded, controlled study.	Oral colistin (2× 106 IU 4× daily) 14 days	31:31 Patients with haemotologi-cal malignancies. Carriers of multidrug-resistant/extensively drug-resistant Gram-negative bacteria [21].	49 (IQR 36–63)	21 days	19/31 in the intervention group, 10/31 in the control group.	13/31 in the intervention group, 12/31 in the control group.
Dimitriou et al. [17] 2019 Germany	Randomised (2:1), double-blinded placebo-controlled multi-centre study.	Oral colistin (2× 106 IU 4× daily) and gentamicin (80 mg 4× daily) for 7 days, and 3 doses of fosfomycin (3 g every 72 h)	18:11 Severely immuno-compromised patients. Carriers of ESBL-E.	52 (30–73) in the intervention group, 53 (28–64) in the placebo group	28 and 42 days	11/18 (61.1%) in the intervention group versus 2/11 (18.2%) in the placebo group	7/18 (38.9%) in the intervention group versus 3/11 (27.3%) in the placebo group
Farinas et al. [19] 2021 Spain	Randomised (1:1), open label, parallel-group, controlled multi-centre study.	Oral colistin (1.26× 106 IU 4× daily) and neomycin (178 mg 4× daily) for 14 days.	53:52 Patients undergoing solid organ transplantation. Carriers of ESBL, AmpC or carbapenemase-producing *Enterobacterales*	56.3 (SD 11.0) in the interven-tion group, 57.0 (SD 12.6) in the control group	30 days	Not available	24/53 in the intervention group versus 14/52 in the control group

## Supplementary Materials

The following supporting information can be downloaded at: https://www.mdpi.com/article/10.3390/antibiotics13080747/s1, Figure S1: The funnel plot from the meta-analysis; Figure S2: The results of the risk of bias analysis; Document S1: The full search strategy from the EMBASE, Cochrane Central and PubMed databases; Document S2: The Quality Assessment Tool from the National Institutes of Health.
