# Supplementary material for: The Effect of Antibiotics on the Eradication of Multidrug-Resistant Organisms in Intestinal Carriers—A Systematic Review with Meta-Analysis
Source: Antibiotics (Basel). 2024 Aug 9;13(8):747. doi: 10.3390/antibiotics13080747 (PMC11350669; doi:10.3390/antibiotics13080747)
Supplement: Supplementary file 1 [file antibiotics-13-00747-s001.zip › Supplementary document 1.pdf]

PubMed:

(((((((((((((((((((((((((((Vancomycin Resistance[MeSH Terms]) OR (Vancomycin-Resistant Enterococci[MeSH Terms])) OR (Drug Resistance, Multiple[MeSH Terms])) OR (Carbapenem-Resistant Enterobacteriaceae[MeSH Terms])) OR (Vancomycin resistan*[Text Word])) OR (Antibiotic resistan*[Text Word])) OR ("extended spectrum beta lactamase"[Text Word])) OR ("extended-spectrum beta-lactamase"[Text Word])) OR ("extended-spectrum beta lactamase"[Text Word])) OR (carbapenemase producing[Text Word])) OR (carbapenemase resistan*[Text Word])) OR (multidrug resistan*[Text Word])) OR (multi-drug resistan*[Text Word])) OR (esbl[Text Word])) OR (cpo[Text Word])) OR (cpe[Text Word])) OR (cre[Text Word])) OR (crkp[Text Word])) OR (kpc[Text Word])) OR (carbapenem-resistan*[Text Word])) OR (carbapenem resistan*[Text Word])) OR (vre[Text Word])) OR (vrefm[Text Word])) OR (carbapenemase-producing[Text Word])) AND ((((((((((((((((((((((((((((((((Anti-Bacterial Agents[MeSH Terms]) OR (colistin[MeSH Terms])) OR (polymyxins[MeSH Terms])) OR (gentamicins[MeSH Terms])) OR (Paromomycin[MeSH Terms])) OR (ceftazidime[MeSH Terms])) OR (meropenem[MeSH Terms])) OR (imipenem[MeSH Terms])) OR (ertapenem[MeSH Terms])) OR (linezolid[MeSH Terms])) OR (daptomycin[MeSH Terms])) OR (tobramycin[MeSH Terms])) OR (neomycin[MeSH Terms])) OR (avibactam, ceftazidime drug combination[MeSH Terms])) OR (Tigecycline[MeSH Terms])) OR (antibiotic*[Text Word])) OR (antibacterial[Text Word])) OR (anti-bacterial[Text Word])) OR (neomycin[Text Word])) OR (colistin[Text Word])) OR (polymyxin[Text Word])) OR (gentamicin[Text Word])) OR (Paromomycin[Text Word])) OR (ceftazidime[Text Word])) OR (meropenem[Text Word])) OR (imipenem[Text Word])) OR (ertapenem[Text Word])) OR (linezolid[Text Word])) OR (daptomycin[Text Word])) OR (tobramycin[Text Word])) OR (ceftazidime-avibactam[Text Word])) OR (Tigecycline[Text Word]))) AND ((((((((((((((((((Enterobacteriaceae[MeSH Terms]) OR ("Gram-Negative Bacteria"[MeSH Terms])) OR (Enterococcus faecium[Text Word])) OR (E faecium[Text Word])) OR (Enterococci[Text Word])) OR (Enterobacteriaceae[Text Word])) OR (Enterobacterales[Text Word])) OR (Escherichia coli[Text Word])) OR (E coli[Text Word])) OR (Klebsiella[Text Word])) OR (Klebsiella pneumoniae[Text Word])) OR (Klebsiella oxytoca[Text Word])) OR (K oxytoca[Text Word])) OR (K pneumoniae[Text Word])) OR ("Gram-negative bacteria"[Text Word])) OR ("Gram negative bacteria"[Text Word])) OR (Enterobacteria[Text Word])) OR (Enterobacteriae[Text Word]))) AND ((((Eradicat*[Text Word]) OR (Decoloniz*[Text Word])) OR (decolonis*[Text Word])) OR ("selective digestive decontamination"[Text Word]))) NOT (helicobacter[MeSH Terms])

Cochrane:

Search Name: MRO decolonization

Date Run: 17/11/2023 19:23:20

Comment:

ID	Search	Hits

#1 MeSH descriptor: [Anti-Bacterial Agents] explode all trees 15306

#2 antibiotic* OR antibacterial OR anti-bacterial OR neomycin OR colistin OR polymyxin OR gentamicin OR Paromomycin OR ceftazidime OR meropenem OR imipenem OR ertapenem OR linezolid OR daptomycin OR tobramycin OR ceftazidime-avibactam OR Tigecycline 50249

#3 "Vancomycin resistance" OR "Antibiotic resistance" OR “extended spectrum beta lactamase” OR “extended spectrum beta lactamase” OR “extended spectrum beta lactamase” OR "carbapenemase producing" OR "carbapenemase resistant" OR "multidrug resistance" OR esbl OR cpo OR cpe OR cre OR crkp OR kpc OR carbapenem resistance OR carbapenem resistant OR vre OR vrefm OR "carbapenemase producing" 5391

#4 "Enterococcus faecium" OR "E faecium" OR Enterococci OR Enterobacteriaceae OR Enterobacterales OR "Escherichia coli" OR "E coli" OR Klebsiella OR "Klebsiella pneumoniae" OR "Klebsiella oxytoca" OR "K oxytoca" OR "K pneumoniae" OR “Gram negative bacteria” OR “Gram negative bacteria” OR Enterobacteria OR Enterobacteriae 7021

#5 Eradicat* OR Decoloniz* OR decolonis* OR decontaminat* OR “selective digestive decontamination” 10856

#6 #1 AND #2 14204

#7 #6 AND #5 2128

#8 #6 AND #5 AND #4 AND #3 63

Embase

#	Query	Results from 20 Nov 2023
1	exp antiinfective agent/	4,653,784
2	(antibiotic* or antibacterial or anti-bacterial or neomycin or colistin or polymyxin or gentamicin or Paromomycin or ceftazidime or meropenem or imipenem or ertapenem or linezolid or daptomycin or tobramycin or ceftazidime-avibactam or Tigecycline).mp. [mp=title, abstract, heading word, drug trade name, original title, device manufacturer, drug manufacturer, device trade name, keyword heading word, floating subheading word, candidate term word]	1,167,862
3	("Vancomycin resistance" or "Antibiotic resistance" or "extended spectrum beta	372,323

	lactamase" or "extended spectrum beta lactamase" or "extended spectrum beta lactamase" or "carbapenemase producing" or "carbapenemase resistant" or "multidrug resistance" or esbl or cpo or cpe or cre or crkp or kpc or carbapenem resistance or carbapenem resistant or vre or vrefm or "carbapenemase producing").mp. [mp=title, abstract, heading word, drug trade name, original title, device manufacturer, drug manufacturer, device trade name, keyword heading word, floating subheading word, candidate term word]	
4	("Enterococcus faecium" or "E faecium" or Enterococci or Enterobacteriaceae or Enterobacterales or "Escherichia coli" or "E coli" or Klebsiella or "Klebsiella pneumoniae" or "Klebsiella oxytoca" or "K oxytoca" or "K pneumoniae" or "Gram negative bacteria" or "Gram negative bacteria" or Enterobacteria or Enterobacteriae).mp. [mp=title, abstract, heading word, drug trade name, original title, device manufacturer, drug manufacturer, device trade name, keyword heading word, floating subheading word, candidate term word]	639,240
5	(Eradicat* or Decoloniz* or decolonis* or decontaminat* or "selective digestive decontamination").mp. [mp=title, abstract, heading word, drug trade name, original title, device manufacturer, drug manufacturer, device trade name, keyword heading word, floating subheading word, candidate term word]	133,637
6	(#1 and #2).mp. [mp=title, abstract, heading word, drug trade name, original title, device manufacturer, drug manufacturer, device trade name, keyword heading word, floating subheading word, candidate term word]	6,534,224
7	(#1 and #3).mp. [mp=title, abstract, heading word, drug trade name, original title, device manufacturer, drug manufacturer, device trade name, keyword heading word, floating subheading word, candidate term word]	5,112,943

8	(#1 and #4).mp. [mp=title, abstract, heading word, drug trade name, original title, device manufacturer, drug manufacturer, device trade name, keyword heading word, floating subheading word, candidate term word]	4,097,771
9	(#1 and #5).mp. [mp=title, abstract, heading word, drug trade name, original title, device manufacturer, drug manufacturer, device trade name, keyword heading word, floating subheading word, candidate term word]	3,856,794
10	(#11 and #12 and #13 and #14).mp. [mp=title, abstract, heading word, drug trade name, original title, device manufacturer, drug manufacturer, device trade name, keyword heading word, floating subheading word, candidate term word]	87,758
11	exp animal/ not exp Human/	5,168,627
12	exp "Helicobacter pylori"/	62,389
13	10 not 11	85,156
14	13 not 12	85,017
15	2 and 3 and 4 and 5	1,983
16	15 not 11	1,808
17	16 not 12	1,753
