# Supplementary material for: The Effect of Antibiotics on the Eradication of Multidrug-Resistant Organisms in Intestinal Carriers—A Systematic Review with Meta-Analysis
Source: Antibiotics (Basel). 2024 Aug 9;13(8):747. doi: 10.3390/antibiotics13080747 (PMC11350669; doi:10.3390/antibiotics13080747)
Supplement: Supplementary file 1 [file antibiotics-13-00747-s001.zip › Supplementary document 2 legend.pdf]

Supplementary document S2 contains the Quality Assessment Tool from the National Institutes of Health (https://www.nhlbi.nih.gov/health-topics/study-quality-assessment-tools) that we used in a modified version to perform the risk of bias analysis.
