# Supplementary material for: The Effect of Antibiotics on the Eradication of Multidrug-Resistant Organisms in Intestinal Carriers—A Systematic Review with Meta-Analysis
Source: Antibiotics (Basel). 2024 Aug 9;13(8):747. doi: 10.3390/antibiotics13080747 (PMC11350669; doi:10.3390/antibiotics13080747)
Supplement: Supplementary file 1 [file antibiotics-13-00747-s001.zip › Supplementary figure 2 legend.pdf]

Supplementary Figure S2 shows the results from the risk of bias analysis. We coloured the criteria with low risk of bias green, the criteria with unclear risk of bias black, and the criteria with high risk of bias red.
