# Supplementary material for: The Effect of Antibiotics on the Eradication of Multidrug-Resistant Organisms in Intestinal Carriers—A Systematic Review with Meta-Analysis
Source: Antibiotics (Basel). 2024 Aug 9;13(8):747. doi: 10.3390/antibiotics13080747 (PMC11350669; doi:10.3390/antibiotics13080747)
Supplement: Supplementary file 1 [file antibiotics-13-00747-s001.zip › Supplementary figure 2.pdf]

	Saidel-Odes et al. 2012	Huttner et al. 2013	Stoma et al. 2018	Dimitriou et al. 2019	Farinas et al. 2021
RCT					
Adequate method of randomisation					
Concealment of treatment allocation					
Blinding of participants and providers					
Blinding of people assessing outcome					
Baseline similarities					
Drop out of 20% or less					
Differential drop-out rate					
Adherence/compliance by treatment group					
Similar background treatments					
Outcome assessment reliability					
Power and sample size					
Prespecification of outcomes and subgroups/No subgroup analysis					
Analysis in other groups than intended					
Duration of treatment specified					
Antibiotic/dosage of treatment specified					
