# Supplementary figures and images for: The Effect of Antibiotics on the Eradication of Multidrug-Resistant Organisms in Intestinal Carriers—A Systematic Review with Meta-Analysis
Source: Antibiotics (Basel). 2024 Aug 9;13(8):747. doi: 10.3390/antibiotics13080747 (PMC11350669; doi:10.3390/antibiotics13080747)

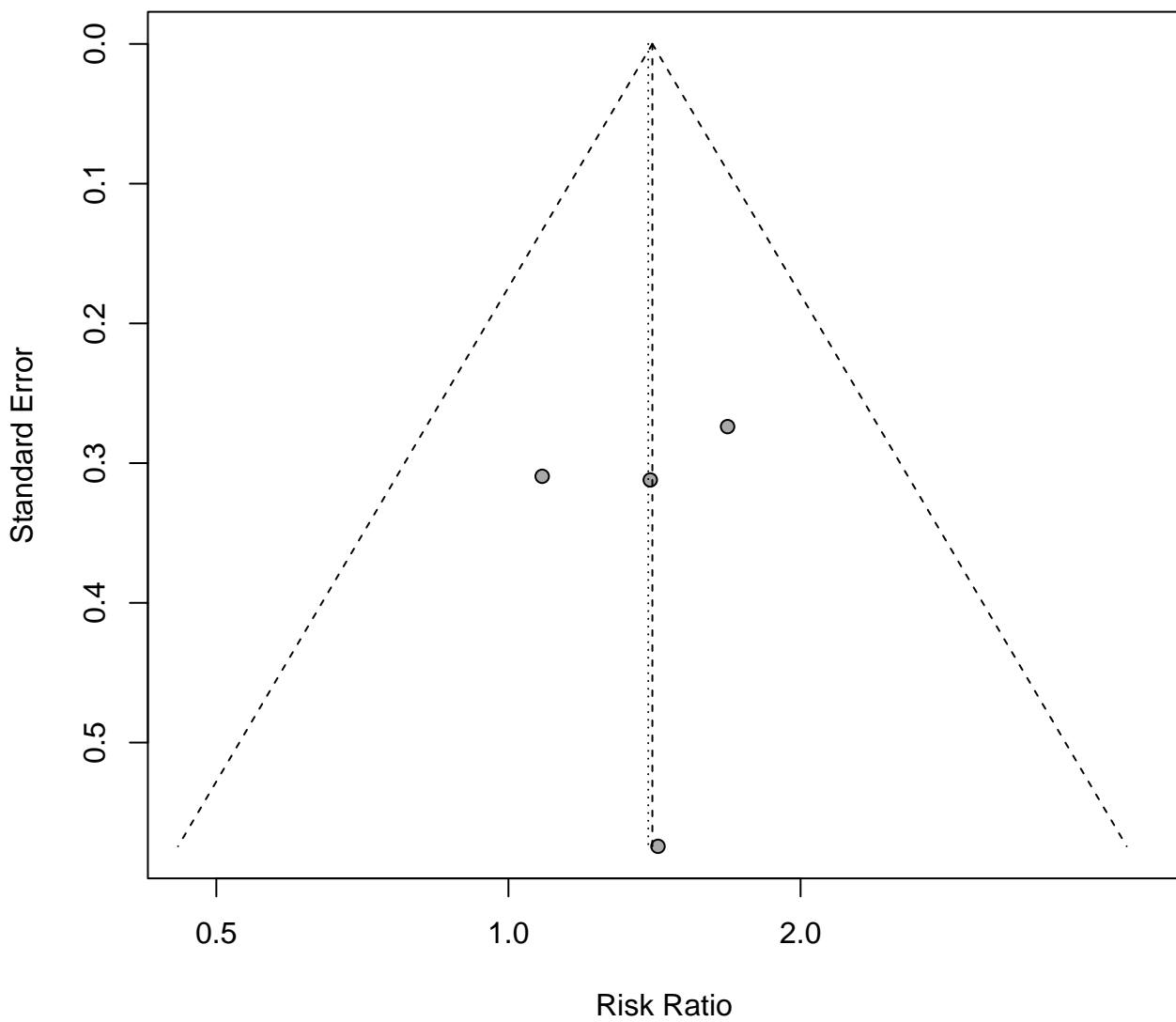

Supplement: Supplementary file 1 [file antibiotics-13-00747-s001.zip › Supplementary figure 1.pdf]
